# Digital support for quality assurance in 24-hour caregiving at home: a randomized controlled trial investigating the effects on quality of life and professional skills of paid 24h-caregivers

**DOI:** 10.1186/s12877-023-04454-4

**Published:** 2023-11-17

**Authors:** Elisabeth Haslinger-Baumann, Peter Putz, Carina Hauser, Elisabeth Kupka-Klepsch, Nadine Sturm, Franz Werner

**Affiliations:** 1 FH Campus Wien University of Applied Sciences, Favoritenstraße 226, 1100 Vienna, Austria; 2 Johanniter Österreich Ausbildung und Forschung gemeinnützige GmbH, Ignaz-Köck-Straße 22, 1210 Vienna, Austria

**Keywords:** 24-h home care, e-learning, Digital care documentation, Active and assisted living, COVID-19

## Abstract

**Background:**

Regarding the care of older adults, 24-h home-care represents a cornerstone, with > 32,000 service users in Austria. Our research project *24hQuAALity* aimed to develop and evaluate a distributed client-server software solution for the support and quality assurance of this home-care service. In this trial, we investigated the effects of this intervention on the quality of life and professional skills of paid 24h-caregivers in Austria.

**Methods:**

The application used in our study comprises an e-learning platform, an integrated emergency management, networking opportunities, and an electronic care documentation system in the native language of the 24h-caregivers. The trial was conducted using a parallel three-arm study design to evaluate (i) a control group, which performed usual home care, (ii) a partial intervention group, which used the e-learning and networking platforms, and (iii) a full intervention group, which used the entire intervention (e-learning platform, networking platform, and digital care documentation). Primary self-reported outcomes were the standardized *ASCOT for Carers* score and a score based on responses to project-specific efficacy questions.

**Results:**

Among the 110 24h-caregivers who were randomly classified into the three groups, *ASCOT for Carers score* data were available for 57 and 35 24h-caregivers at 5- and 9-month follow-up examinations, respectively. At 9 months, 24h-caregivers receiving any intervention rated the *ASCOT for Carers score* (not significantly) better than the controls (*p* = 0.05, η_p_^2^ = 0.15), mainly in the domain “feeling encouraged and supported”. At 9 months, 24h-caregivers receiving any intervention rated the project-specific *Efficacy score* significantly better than the controls (*p* = 0.02, η_p_^2^ = 0.20), mainly due to better ratings in the subitems “satisfaction with current docu”, “docu supports doing my job”, “ I’m well prepared for emergencies”, “my professional skills are adequate for doing my job”, and “communication with contacts”.

**Conclusions:**

Providing e-learning and e-documentation devices to 24h-caregivers improved their care-related quality of life, mainly because they felt more encouraged and supported. Moreover, these interventions improved their self-perceived professional skills. As an extrapolation of findings, we found that these interventions could empower 24h-caregivers and improve the quality of home-care services provided by them.

**Trial registration:**

Digital Support for Quality Assurance in 24-h Caregiving at Home was registered and posted on the ClinicalTrials.gov public website (ClinicalTrials.gov Identifier: NCT04581538).

**Supplementary Information:**

The online version contains supplementary material available at 10.1186/s12877-023-04454-4.

## Background

Owing to demographic changes, the demand for professional and institutional care, as well as 24-h home care is increasing worldwide. In several European countries, migrant care work in private households has become an important, but often illegal, source of long-term care provision. The 24-h home care reform in Austria in 2007 was a comprehensive attempt to regularize previously illegal arrangements [1], whereas such attempts have been less comprehensive in other European countries, such as Germany, Italy, and Spain [2]. Moreover, owing to its cost-efficiency and availability, paid 24-h home care has been recognized as a cornerstone in the care of older adults. Furthermore, with > 32,000 users in Austria [3], it is an important alternative to family assistance and mobile care.

The offered 24-h care ranges from assistance in household activities to round-the-clock care. The system is financially supported by a mix of private and public funding. The prerequisite for receiving public funds (up to 1280€ per month) is that care allowance is received for at least care level three and that an income limit is not exceeded. Regarding qualifications, the prerequisite for obtaining funding is theoretical training equivalent to that of a home care assistant or ≤ 6 months of proper care of the person in need. However, as known from previous studies [2], a few 24h-caregivers are even graduate nurses.

Notably, < 2% of the 24h-caregivers are from Austria [4]; therefore, they usually commute between Austria and their home countries (mainly Slovakia, Romania, and Hungary) once every ≥ 2 weeks [2]. As these 24h-caregivers do not have a permanent residence permit, they have to interrupt their work visits. Additionally, stressful working conditions primarily arise from language problems and living in isolation with a person (often affected by dementia) in combination with other factors, such as little or no relevant professional training and quality control.

The randomized controlled trial of the *Austrian RegionAAL study*, which evaluated a program of interventions (such as medications, drinking and physical activity reminders, automatic light systems, watches to detect falls, or video telephony with caregivers), reported no improvement in anxiety levels among caregivers, but a tendency to reduce some aspects of burdens [5]. Moreover, the importance of quality assurance in 24-h home care was highlighted by the *Austrian Institute of Economic Research* in 2017 [4]. The core issues that were identified included consideration of the interests of 24h-caregivers and quality of life as well as the duty of service documentation. Specific actions require delegation or professional training from a certified nurse or another healthcare professional. At present, only minor regular examinations are performed to determine compliance with quality standards. Despite the Austrian legislative reform in 2007, 24-h home care remains a poorly regulated branch that, from a legal perspective, lacks standards for the delivery of the service, leading to burdensome working conditions for 24h-caregivers [6].

We conducted the research project *24hQuAALity* to develop and evaluate a distributed client–server software solution for the support and quality assurance of 24-h home care. The present trial investigated the effects of this intervention on the quality of life and professional skills of paid 24h-caregivers in Austria.

## Methods

### Trial design, setting, and participants

The present study followed a parallel three-arm study design in which the households were evenly distributed. The three parallel arms evaluated a (i) control group, who continued usual home care (including unstructured paper–pencil documentation), (ii) a partial intervention group, which used e-learning and networking platforms, and (iii) a full intervention group, which used the entire intervention (e-learning platform, networking platform, and digital care documentation). The framework was designed to test for the superiority of the intervention. Outcomes were assessed at baseline and two follow-up assessments were planned at 3 and 12 months, but these were actually performed at an average of 5 and 9 months, respectively. A household was considered eligible for inclusion if (i) the household was currently receiving a 24-h-care service, (ii) the household was located in the federal territory of Austria, (iii) the 24h-caregiver was able and willing to comply with all study-related procedures and provided informed consent, and (iv) the care receiver was aged ≥ 55 years. In contrast, households were excluded if (i) the care receiver had died, (ii) 24-h care was terminated for reasons other than the death of the care receiver, and (iii) 24-h care was interrupted for ≥ 8 weeks. No changes were made to the eligibility criteria or trial outcomes after starting the trial. This paper has been written as per the CONSORT checklist of information to be included for reporting a randomized trial [7].

In a mixed-methods approach that integrates (i) investigator observations, (ii) interviews with 24h-caregivers, (iii) interviews with relatives, and (iv) interviews with care receivers (where possible), trained investigators should provide reliable ratings on the care receivers' quality of life via triangulation [8]. Owing to the coronavirus disease 2019 (COVID-19) pandemic-related restrictions, the entire data collection was adapted to online surveys (EFS Survey, Tivian XI GmbH, Cologne, and Germany). Only data from the target group of 24h-caregivers had sufficient responses to conduct a quantitative analysis. Notably, sample size calculation was based on the effects on the quality of life of care receivers. Overall, 168 households were targeted for the survey. Further, considering an anticipated dropout rate of 20%, 135 households were expected to complete all study-related procedures. With an alpha of 0.05 and a beta of 0.20 (power: 0.80) and an effect size f of 0.3, the sample size of 45 households was estimated for each of the three study arms. This effect size f of 0.3 was assumed based on estimation, and this represented a medium-sized effect [9].

### Stratified randomization and recruitment

Stratified randomization was applied based on the care receiver’s care level and 24h-caregiver’s professional experience (see scoring scheme in Supplementary Table S1) to achieve balanced treatment allocation among these covariates (see supplementary material for randomization details). Moreover, assistance in completing the survey (e.g., in opening the survey link) was offered by the study recruitment team during face-to-face attendance as needed. In addition, language barriers were overcome by offering the survey in the 24h-caregivers’ native languages.

Participants were recruited by four project partner organizations, namely *Caritas Rundum Betreut, Home-Care-Management Alexander Winter*, *Institut für Personenbetreuung,* and *Johanniter Forschung*. Recruitment strategies were recruitment of the organizations’ customers, recruitment at organizational level, and recruitment via individual 24h-caregivers. Individual survey links were distributed via email, WhatsApp, or Facebook messengers. The recruiting project partners delivered the tablets to the households. Using training materials, they trained the 24h-caregivers in using the software. The training materials included a quick start guide and, for the full intervention group only, an e-documentation manual. In addition, explanatory videos were available on the home screen of the tablets. Author PP generated the random allocation sequence. Due to the nature of the intervention, no measures were taken to blind participants, nor were outcome assessors blinded.

All 24h-caregivers provided written informed consent before participation. Ethical approval was obtained from the ethics committee of the Evangelical Hospital Vienna (EC Number: 012019). The trial was registered at ClinicalTrials.gov under the identifier NCT04581538. Households were enrolled between October 2020 and March 2021.

### Interventions

Based on the results of a qualitative requirements study, emergency management skills and information about common geriatric conditions and legal aspects of 24-h care were identified as the main needs of the 24h-caregivers [10]. The application developed in the project *24hQuAALity* comprises the following features: (i) an information and education portal (e-learning platform) with interactive learning content (33 courses) about common diseases and short videos on recurrent 24h-home-caregiving situations (on the topics of work settings, legal principles for 24-h caregiving (e.g., delegation), German language training, emergency skills, geriatric diseases, and nursing care as well as measures in daily care and housekeeping); (ii) a comprehensive electronic care documentation that supports quality assurance and ensures transparency between the involved individuals. The electronic care documentation is individually adjustable according to the diseases of the clients. It is mainly completed by checkboxes and the remaining free-text fields are supported by a translation function; (iii) an integrated emergency management, enabling 24h-caregivers to react quickly and professionally to emergencies; and (iv) a networking platform providing links to translation pages or networking opportunities with members and relatives. The networking platform is facilitated via a moderated Facebook group and the messenger service Signal.

The intervention was administered via a device (a tablet with the client–server software solution and instructions for use until follow-up 2) in German as well as in Slovak, Hungarian, and Romanian – the most common languages spoken by the 24h-caregivers. Intervention group participants received online certificates for each completed e-learning course.

### Outcome measures

The primary prespecified outcome measure used in this study was the metric *ASCOT for Carers* [11] score (henceforth *ASCOT score*) that summarizes seven items [12] on a scale of 0–1, with 1 indicating the most positive outcome. The seven attributes are related to the informal caregivers’ social care-related quality of life. The individual *ASCOT* items are self-rated on a 4-point Likert scale, with 1 and 4 indicating “ideal” and “high needs,” respectively. In addition to the standardized *ASCOT* measure, 14 project-specific efficacies for 24h-caregivers’ items were self-rated on a 5-point Likert scale, with 5 indicating the most positive outcome. These items (including those related to documentation, professional skills, feeling competent, and social/professional interaction) were based on the specific benefits expected from the intervention. A mean metric score was calculated on a scale of 0–1, where 1 indicated the most positive outcome (see supplementary material for score formulas and complete item specifications). Additionally, statistics were reported for each subitem of the scores to enable contextual interpretation. Except for a content validation approach, where feedback was obtained from project team members, no information is available on the reliability and validity of the *Efficacy score* and its subitems.

For sample characterization, the following baseline characteristics were collected: for care receivers: (i) age (metric), (ii) sex (nominal), and (iii) care level (ordinal); and for 24h-caregivers: (iv) age (metric), (v) professional home-care experience (metric), (vii) any care education (nominal), and (viii) nationality (nominal).

The *ASCOT* was identified as the best available standardized assessment, based on the logical assumption that specific education leads to a higher level of safety in doing the job and that consequent higher job satisfaction triggers an improvement in care-related quality of life. Specific enhancement domains expected from the interventions were identified in advance by the project team: quality of assistance, health-related quality of life, perceived safety, efficiency, and resilience. The *ASCOT* does not fully correspond to these areas, and thus the aforementioned set of project-specific questions addressing these domains were added as outcome measures. A second follow-up was planned to gain insights into the sustainability of effects, for which no a priori assumptions were made.

### Statistical methods

Survey responses were analyzed in the statistical software SPSS version 27 (IBM Corp., 2021 Armonk, NY). Planned comparisons were conducted between the control group and any intervention group (contrast 1) and between the partial and full intervention groups, which additionally used the digital nursing documentation (contrast 2). Thus, contrasts 1 and 2 tested for any effect of the intervention and whether the full intervention was additionally beneficial, respectively.

Metric baseline characteristics were expressed as mean values with standard deviations. Categorical baseline characteristics were presented as frequencies (n, %). Unadjusted outcome scores were reported as mean values with 95% confidence intervals (95% CI) (see Supplementary Tables). To allow for graphical data inspection, *ASCOT score* and *Efficacy score* are provided per study arm (test group, partial intervention, full intervention) as beeswarm plots and corresponding boxplots at both follow-ups (see Supplementary Figures).

Treatment effects at two follow-up periods were planned to be tested using one-way analysis of variance (ANOVA). However, in accordance with a prespecified approach, analysis of covariance (ANCOVA) was conducted to correct for the baseline imbalance detected. The respective baseline variable was used as the covariate. As an additional explorative analysis, we ran the ANCOVA with “nested data” as a second covariate. *Nested* means in this case that 2 participating 24h-caregivers worked consecutively in the same household. Beyond that, no further adjustments for covariates or additional analyses were performed. The overall ANCOVA results were independently reported for follow-ups 1 and 2 as test statistic F, *p*-value, and ηp^2^ (partial eta^2^), interpreted according to Cohen’s scheme, where 0.01, 0.06, and 0.14 represented small, medium, and large effect sizes, respectively [9]. Shapiro–Wilk tests and additional graphical inspections of q–q plots were applied to test for the normality assumption. The additional ANCOVA assumption of the covariate being equal across the groups was assessed via one-way ANOVA and the homogeneity of the regression slopes with the interaction term factor*covariate.

All descriptive statistical values and corresponding numbers of subjects (n) were reported with mentioning of losses to follow-ups. Effect sizes were calculated and reported accordingly (per protocol). Additionally, overall hypotheses of the *ASCOT* and *Efficacy scores* were analyzed according to the intention-to-treat principle (last value carried forward), which provides a more conservative effect estimate [13], when missing observations introduce bias.

Alpha was set at 0.05, and exact *p*-values were reported. *P*-values were corrected with the Bonferroni-Holm method, which means in the particular case, that the smaller of two contrast *p*-values was multiplied by two, and the larger one remained unchanged, thus correcting for the multiplicity of testing within each hypothesis model.

## Results

### Participants

**Fig. 1:**
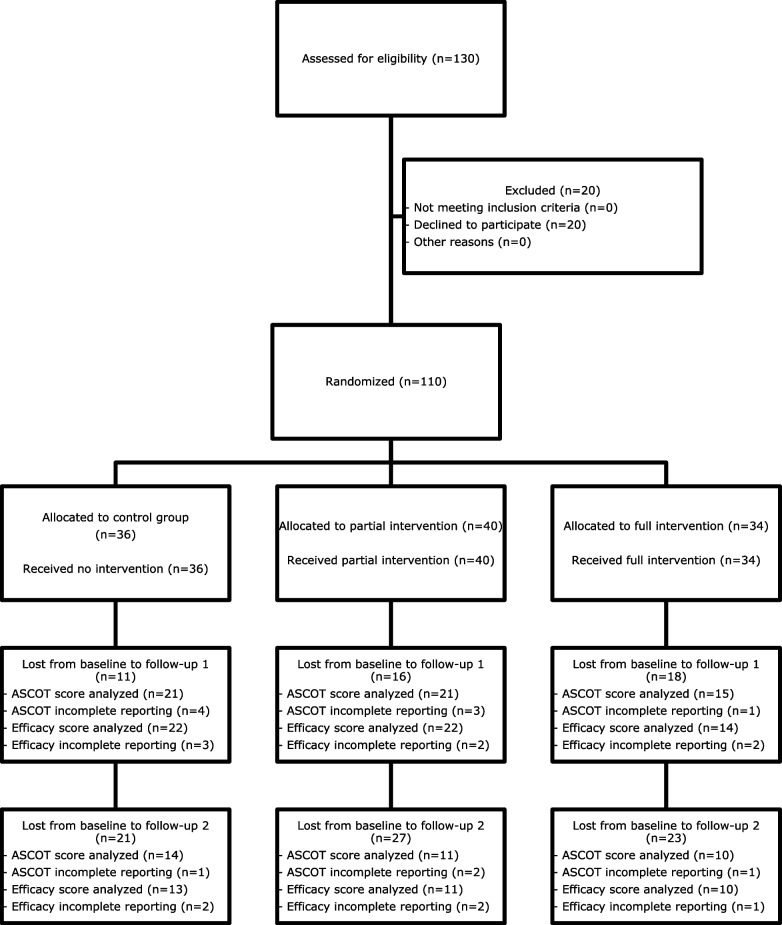
Participant flow diagram

Overall, 110 24h-caregivers were randomly assigned to the three study arms. The data of *ASCOT scores* were available for 57 and 35 24h-caregivers at follow-ups 1 and 2, respectively. Regarding follow-up 1, these 57 24h-caregivers worked in 45 households, because there were 12 households where 2 24h-caregivers worked consecutively. Regarding follow-up 2, these 35 24h-caregivers worked in 30 households, because there were 5 households where 2 24h-caregivers worked consecutively. *Efficacy score* data were available for 58 and 34 24h-caregivers at follow-ups 1 and 2, respectively (Fig. 1). The main reasons for dropout were the termination of 24-h care and death of the client. The mean age of the 24h-caregivers was 50 years, and 88% of the 24h-caregivers were women. In the partial intervention group, the proportion of women was higher, and those who had completed any care education were under-represented (Table 1). As individual 24h-caregivers commute to Austria once every ≥ 2 weeks for care work, the actual length of stay is likely to have been approximately 50% of the actual study duration and may have been less if there were fluctuations.

**Table 1:** Baseline demographic characteristics

	Control	Partial intervention	Full intervention	Total
**Caregivers**				
Age [years], mean (SD)	48.3 (11.9), *n* = 34	50.8 (10.3), *n* = 40	49.9 (9.3), *n* = 34	49.7 (10.5), *n* = 108
Female sex, n (%)	30 (83.3%)	38 (95.0%)	28 (82.4%)	96 (88.1%)
Professional experience [years], mean (SD)	9.1 (4.9), *n* = 34	9.6 (4.5), *n* = 39	10.5 (7.6), *n* = 32	9.7 (5.7), *n* = 105
Any care education completed, n (%)	35 (97.2%)	36 (90.0%)	33 (97.1%)	104 (94.5%)
**Care receivers**				
Age [years], mean (SD)	84.5 (8.9), *n* = 34	89.3 (7.2), *n* = 39	87.0 (7.7), *n* = 30	87.1 (8.1), *n* = 103
Care level, median (IQR)	4.0 (2.0), *n* = 22	4.0 (2.0), *n* = 22	4.0 (2.0), *n* = 23	4.0 (2.0), *n* = 67

*SD* Standard deviation, *IQR* Interquartile range

The trial was stopped after a one-month extension. The outcome data were collected over 13 months between October 01, 2020, and October 31, 2021. The actual follow-up duration was recorded from the survey completion data, where the aforementioned turnover and fluctuation introduced variation in the actual times to follow-ups. The follow-up 1 assessment was actually completed after a mean duration of 152 days (5 months; median: 146; range: 49–278 days). The follow-up 2 assessment was completed after a mean duration of 273 days (9 months; median: 286; range: 181–348 days).

The e-learning platform was actively used by 63 out of 74 participants who received personalized access via study participation in the partial or full intervention group. The 11 non-users are part of the participant fraction “lost to follow-up 1”. 19 (30%) of the users completed all 33 courses offered, 17 (27%) completed 10 to 32 courses, and 27 (43%) completed 1 to 10 courses. 36 participants of the partial intervention group completed 737 courses during 629 logins. 27 participants of the full intervention group completed 507 courses during 333 logins.

### ASCOT

**Fig. 2:**
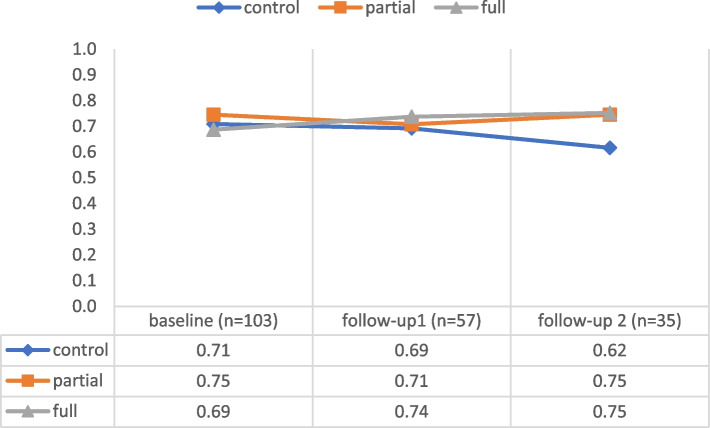
ASCOT score trend

Data analysis revealed some extent of baseline imbalance in the means of the outcome variable *ASCOT score*: control, 0.71 (95% CI: 0.65, 0.77; *n* = 34); partial intervention, 0.74 (95% CI: 0.70, 0.78; *n* = 36); full intervention, 0.69 (95% CI: 0.63, 0.74; *n* = 33). Thus, the hypotheses were tested using ANCOVA, with the baseline variable used as the covariate. Figure 2 shows a negative trend from baseline to follow-up 2 in the control group, whereas the mean scores were maintained or enhanced in the intervention groups (see Supplementary Table S2 for exact values and confidence intervals). The main findings of the inferential statistical analysis are summarized below.

#### Follow-up 1

At 5 months, baseline imbalance corrected means did not significantly differ among the three groups [F(2) = 2.25, *p* = 0.12, ηp^2^ = 0.08, achieved power = 0.47]. Regarding the analysis by the intention-to-treat principle, the effect remained similar [F(2) = 2.52, *p* = 0.09, ηp^2^ = 0.05]. No statistically significant differences in the *ASCOT score* and its subdomains were observed between the control and any intervention groups (Supplementary Table S4). However, the 24h-caregivers in the full intervention group rated the *ASCOT score* (not significantly) better (*p* = 0.07, ηp^2^ = 0.13) than those in the partial intervention group, mainly due to better ratings in the subdomains “self-care” and “space and time to be yourself” (Supplementary Table S5).

#### Follow-up 2

At 9 months, baseline imbalance corrected means did not differ significantly among the three groups [F(2) = 2.76, *p* = 0.08, ηp^2^ = 0.15, achieved power = 0.56]. Regarding the analysis by the intention-to-treat principle, the observed effect was statistically significant [F(2) = 3.14, *p* = 0.047, ηp^2^ = 0.06]. The comparison between the control and any intervention groups revealed that the intervention groups had statistically significant (*p* = 0.03) better ratings, explaining 15% of the variance in the model. Among the subdomains, the item “feeling encouraged and supported” was rated significantly (*p* < 0.01) better by the intervention groups than by the control group, explaining 24% of the variance in the model (Supplementary Table S6). However, no significant effects were observed between 24h-caregivers in the full and partial intervention groups (Supplementary Table S7).

### Efficacy survey

**Fig. 3:**
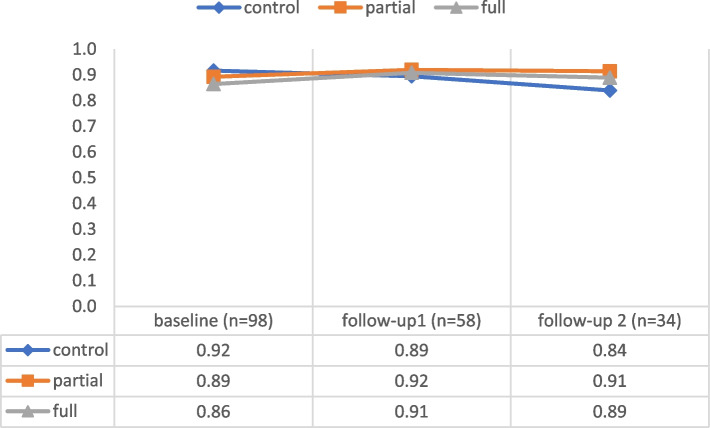
Efficacy score trends

Consistent with the *ASCOT score*, data analysis revealed some extent of baseline imbalance in the means of the outcome variable *Efficacy score*: control, 0.92 (95% CI: 0.88, 0.96; *n* = 33); partial intervention, 0.89 (95% CI: 0.85, 0.93; *n* = 33); and full intervention, 0.86 (95% CI: 0.81, 0.91; *n* = 32). Thus, the hypotheses were tested using ANCOVA, with the baseline variable used as the covariate. Figure 3 shows a negative trend from baseline to follow-up 2 in the control group. However, the mean scores showed a positive trend in the intervention groups (see Supplementary Table S3 for exact values and confidence intervals). The main findings of the inferential statistical analysis are summarized below.

#### Follow-up 1

At 5 months, baseline imbalance corrected means did not differ significantly among the three groups [F(2) = 0.42, *p* = 0.66, ηp^2^ = 0.02, achieved power = 0.12]. Regarding the analysis by the intention-to-treat principle, the effect remained similar [F(2) = 0.50, *p* = 0.61, ηp^2^ = 0.01]. No statistically significant differences in the *Efficacy score* and its subdomains were observed between the control and any intervention groups (Supplementary Table S8). The same result was observed in the comparison between the partial and full intervention groups, except for the subitem “satisfaction with current docu,” where the full intervention group had a worse mean rating, however explaining only 5% of the variance in the model (Supplementary Table S9).

#### Follow-up 2

At 9 months, baseline imbalance corrected means differed significantly among the three groups [F(2) = 3.96, *p* = 0.03, ηp^2^ = 0.21, achieved power = 0.72]. However, this was not confirmed by the intention-to-treat analysis [ F(2) = 1.84, *p* = 0.17, ηp^2^ = 0.04]. The comparison between the control and any intervention groups revealed a statistically significant (*p* = 0.02) better score in the intervention groups, explaining 20% of the variance in the model. At least medium-sized effects (ηp^2^ > 0.06) were observed for the subitems “satisfaction with current docu”, ”docu supports doing my job”, “ I’m well prepared for emergencies”, “my professional skills are adequate for doing my job”, and “communication with contacts” with all items in favor of the intervention groups (Supplementary Table S10). No significant additional effects were observed for 24h-caregivers in the full intervention group compared with those in the partial intervention group (Supplementary Table S11).

Additional explorative ANCOVA analyses accounting for the nested data structure (when two 24h-caregivers worked consecutively in the same household) of the *ASCOT* score and the *Efficacy score*, resulted in slightly smaller F-values but did not alter any interpretation in terms of statistical significance (results shown in the captions of Supplementary Tables S4-S11).

## Discussion

Our research project *24hQuAALity* aimed to develop and evaluate a distributed client–server software solution for the support and quality assurance of this care. In the present trial, we investigated the effects of this intervention on the quality of life and professional skills of paid 24h-caregivers in Austria. Both the *ASCOT* and *Efficacy scores* are on a scale of 0–1, with 1 indicating the most positive outcome*. ASCOT score* results ranged from 0.62 to 0.75 across the groups and time points studied. Among the *ASCOT* subitems, “personal safety” was rated best at baseline, whereas the highest needs were identified for the subitems “space and time to be yourself” as well as “control over daily life”. *Efficacy score* results ranged from 0.84 to 0.92 across the groups and time points studied. As the *Efficacy score* values were already high at baseline, there was limited room for improvement in this metric. For both the *ASCOT* and *Efficacy scores*, a negative trend from baseline to follow-up 2 was observed in the control group, whereas the mean scores were enhanced or maintained in the intervention groups. At 9 months, 24 h-caregivers receiving any intervention rated the *ASCOT score* significantly better than the control group, mainly due to better ratings in the domain “feeling encouraged and supported” where the effect sizes were large. No additional effect was observed in the full intervention group (including the digital care documentation) at 9 months. However, the partial intervention group reported a worse *ASCOT* score at 5 months. At 9 months, 24h-caregivers receiving any intervention rated the *Efficacy score* significantly better than the control group, mainly due to better ratings in the subitems “satisfaction with current docu”, “docu supports doing my job”, “I’m well prepared for emergencies”, “my professional skills are adequate for doing my job”, and “communication with contacts” where the effects were at least medium-sized. 24h-caregivers who received the full intervention (including the digital care documentation) exhibited a tendency toward rating their satisfaction with the current documentation worse. A prespecified additional intention-to-treat analysis enabled appraisal of whether attrition enhanced the effects. This appraisal suggests that the effects observed after 9 months were partly caused by attrition, making the more conservative intention-to-treat measures more reliable, thereby reducing the observed effect sizes to medium (*ASCOT score*) and small (*Efficacy score*).

In the present study, we observed a trend toward improved social care-related quality of life and project-specific items on self-perceived professional skills, feeling competent, and social/professional interaction. In an extrapolation of these findings to burden, they support the trend toward reduced aspects of burden, as reported in the *Austrian RegionAAL* study [5]. These aspects are important for the 24h-caregivers’ work in private home settings. 24-h caregiving situations are often overwhelming, both professionally and personally. Language problems further exacerbate the situation as the 24h-caregivers come from Eastern Europe, and German is not their native language [3]. Significant improvements in the areas of feeling supported and encouraged may enhance the daily home care routine by supporting successful care arrangements between 24h-caregivers and clients. This can increase the quality of life for all individuals involved and contributes to an improvement in the quality of care.

### Limitations

This study reported results of interventions addressing the quality of life and self-perceived professional skills of 24h-caregivers. The trial was designed to primarily examine the *ASCOT score* of eight *ASCOT* domains related to care receivers. However, owing to the adaptation to an online survey, surveys were analyzed only for 24h-caregivers, where the sample size allowed for quantitative analysis. The *ASCOT for Carers* measure used in this study to assess outcomes among 24-h paid 24h-caregivers was developed for informal caregivers, such as friends or family members. Except for a content validation approach there is no information on the reliability and validity of the *Efficacy score* and its subitems. Fewer households than planned were included. The sample size was approximately 25% of the planned size, which limited the statistical power and the generalizability of the results. The observed effects were therefore interpreted with a particular focus on effect sizes. With the planned sample size, presumably, no baseline imbalance correction would have been necessary. Owing to the nature of the intervention, no measures were taken for blinding of participants, and many participants were lost from the baseline to follow-ups, introducing risk of performance and attrition bias. Although scheduled for 3 and 12 months, the mean time to follow-up was in fact 5 and 9 months, respectively, demonstrating a wide range due to changing work placements and staff turnover. The changed follow-up periods limited the intended interpretation of short- vs. long-term effects. The actual length of stay may have been approximately 50% of the actual study duration or less if there was fluctuation. Baseline-corrected effect size estimates were calculated for all subitems of the scores. When interpreting these results, the focus should be on effect sizes, and the variety of analyses should be considered when interpreting the *p*-values.

## Conclusions

Providing e-learning and e-documentation devices to 24h-caregivers improved their social care-related quality of life, mainly because they felt more encouraged and supported. Moreover, the interventions led to improved self-perceived professional/communication skills, satisfaction with the documentation used, and readiness for emergencies. Independently of the provided e-learning and e-documentation, 24h-caregivers self-rated their professional/communicational skills as high. As an extrapolation of findings, these interventions could empower 24h-caregivers and improve the quality of their home-care services.

### Supplementary Information

**Additional file 1:** Stratified randomization – detailed description.* ASCOT score *calculation details. *Efficacy score *calculation details. **Table S1.** Scoring scheme for professional experience (range: 0-5). **Table S2.** *ASCOT score *descriptive statistics (numeric details corresponding to Fig. 2). **Table S3.***Efficacy score *descriptive statistics (numeric details corresponding to Fig 3). **Table S4.** Follow-up 1 (month 5) ANCOVA ASCOT (baseline value as covariate) comparison control vs. any intervention. **Table S5.** Follow-up 1 (month 5) ANCOVA ASCOT (baseline value as covariate) comparison partial vs. full intervention. **Table S6.** Follow-up 2 (month 9) ANCOVA ASCOT (baseline value as covariate) comparison control vs. any intervention. **Table S7.** Follow-up 2 (month 9) ANCOVA ASCOT (baseline value as covariate) comparison partial vs. full intervention. **Table S8.** Follow-up 1 (month 5) ANCOVA efficacy survey (baseline value as covariate) comparison control vs. any intervention. **Table S9.** Follow-up 1 (month 5) ANCOVA efficacy survey (baseline value as covariate) comparison partial vs. full intervention. **Table S10.** Follow-up 2 (month 9) ANCOVA efficacy survey (baseline value as covariate) comparison control vs. any intervention. **Table S11.** Follow-up 2 (month 9) ANCOVA efficacy survey (baseline value as covariate) comparison partial vs. full intervention. **Table S12.** Pearson’s correlations of baseline data with follow-ups for variance inspection for *ASCOT score *and *Efficacy score*. **Figure S1.** Beeswarm plot and corresponding boxplots for the *ASCOT score *at baseline. **Figure S2.** Beeswarm plot and corresponding boxplots for the *ASCOT score *at the 5-month follow-up. **Figure S3.** Beeswarm plot and corresponding boxplots for the *ASCOT score *at the 9-month follow-up. **Figure S4.** Beeswarm plot and corresponding boxplots for the *Efficacy score *at baseline.**Figure S5.** Beeswarm plot and corresponding boxplots for the *Efficacy score *at the 5-month follow-up. **Figure S6.** Beeswarm plot and corresponding boxplots for the *Efficacy score *at the 9-month follow-up.(PDF 473 KB)

## Data Availability

The datasets used and/or analyzed during the current study are available from the corresponding author on reasonable request.

## Abbreviations

ANOVA: Analysis of variance
ANCOVA: Analysis of covariance
ASCOT: Adult social care outcomes toolkit
CI95: 95% Confidence interval
IQR: Interquartile range
RCT: Randomized controlled trial
SD: Standard deviation
ηp^2^: Partial eta^2^ (effect size measure)
