# Supplementary material for: Digital support for quality assurance in 24-hour caregiving at home: a randomized controlled trial investigating the effects on quality of life and professional skills of paid 24h-caregivers
Source: BMC Geriatr. 2023 Nov 17;23:750. doi: 10.1186/s12877-023-04454-4 (PMC10655364; doi:10.1186/s12877-023-04454-4)
Supplement: Supplementary file 1 — Additional file 1: Stratified randomization – detailed description. ASCOT score calculation details. Efficacy score calculation details. Table S1. Scoring scheme for professional experience (range: 0-5). Table S2. ASCOT score descriptive statistics (numeric details corresponding to Fig. 2). Table S3.Efficacy score descriptive statistics (numeric details corresponding to Fig 3). Table S4. Follow-up 1 (month 5) ANCOVA ASCOT (baseline value as covariate) comparison control vs. any intervention. Table S5. Follow-up 1 (month 5) ANCOVA ASCOT (baseline value as covariate) comparison partial vs. full intervention. Table S6. Follow-up 2 (month 9) ANCOVA ASCOT (baseline value as covariate) comparison control vs. any intervention. Table S7. Follow-up 2 (month 9) ANCOVA ASCOT (baseline value as covariate) comparison partial vs. full intervention. Table S8. Follow-up 1 (month 5) ANCOVA efficacy survey (baseline value as covariate) comparison control vs. any intervention. Table S9. Follow-up 1 (month 5) ANCOVA efficacy survey (baseline value as covariate) comparison partial vs. full intervention. Table S10. Follow-up 2 (month 9) ANCOVA efficacy survey (baseline value as covariate) comparison control vs. any intervention. Table S11. Follow-up 2 (month 9) ANCOVA efficacy survey (baseline value as covariate) comparison partial vs. full intervention. Table S12. Pearson’s correlations of baseline data with follow-ups for variance inspection for ASCOT score and Efficacy score. Figure S1. Beeswarm plot and corresponding boxplots for the ASCOT score at baseline. Figure S2. Beeswarm plot and corresponding boxplots for the ASCOT score at the 5-month follow-up. Figure S3. Beeswarm plot and corresponding boxplots for the ASCOT score at the 9-month follow-up. Figure S4. Beeswarm plot and corresponding boxplots for the Efficacy score at baseline.Figure S5. Beeswarm plot and corresponding boxplots for the Efficacy score at the 5-month follow-up. Figure S6. Beeswarm plot and corresponding boxplo [file 12877_2023_4454_MOESM1_ESM.pdf]

Supplementary Material

Digital support for quality assurance in 24-h caregiving at home: a randomized controlled trial investigating the effects on quality of life and professional skills of paid 24h-caregivers

Table of supplementary content

Page 4: Stratified randomization – detailed description

Page 5: *ASCOT score* calculation details

Page 5: *Efficacy score* calculation details

Page 6: Table S1: Scoring scheme for professional experience (range: 0-5)

Page 7: Table S2. *ASCOT score* descriptive statistics (numeric details corresponding to figure 2)

Page 7: Table S3. *Efficacy score* descriptive statistics (numeric details corresponding to figure 3)

Page 8: Table S4. **Follow-up 1** (month 5) ANCOVA **ASCOT** (baseline value as covariate) comparison **control vs. any intervention**

Page 9: Table S5. **Follow-up 1** (month 5) ANCOVA **ASCOT** (baseline value as covariate) comparison **partial vs. full intervention**

Page 10: Table S6. **Follow-up 2** (month 9) ANCOVA **ASCOT** (baseline value as covariate) comparison **control vs. any intervention**

Page 11: Table S7. **Follow-up 2** (month 9) ANCOVA **ASCOT** (baseline value as covariate) comparison **partial vs. full intervention**

Page 12: Table S8. **Follow-up 1** (month 5) ANCOVA **efficacy survey** (baseline value as covariate) comparison **control vs. any intervention**

Page 14: Table S9. **Follow-up 1** (month 5) ANCOVA **efficacy survey** (baseline value as covariate) comparison **partial vs. full intervention**

Page 16: Table S10. **Follow-up 2** (month 9) ANCOVA **efficacy survey** (baseline value as covariate) comparison **control vs. any intervention**

Page 18: Table S11. **Follow-up 2** (month 9) ANCOVA **efficacy survey** (baseline value as covariate) comparison **partial vs. full intervention**

Page 20: Table S12. Pearson’s correlations of baseline data with follow-ups for variance inspection for *ASCOT score* and *Efficacy score*

Page 21: Figure S1: Beeswarm plot and corresponding boxplots for the ***ASCOT score*** at **baseline**

Page 22: Figure S2: Beeswarm plot and corresponding boxplots for the ***ASCOT score*** at the **5-month follow-up**

Page 23: Figure S3: Beeswarm plot and corresponding boxplots for the ***ASCOT score*** at the **9-month follow-up**

Page 24: Figure S4: Beeswarm plot and corresponding boxplots for the ***Efficacy score*** at **baseline**

Page 25: Figure S5: Beeswarm plot and corresponding boxplots for the ***Efficacy score*** at the **5-month follow-up**

Page 26: Figure S6: Beeswarm plot and corresponding boxplots for the Efficacy score at the **9-month follow-up**

Stratified randomization – detailed description

Stratified randomization was applied based on the care level of the care receiver and the professional experience level of the 24h-caregivers in order to achieve balanced treatment allocation among these covariates. Cut-offs were set at the median of care levels and professional experience levels from a subsample of households enrolled before study commencement. Consequently, two dichotomous covariates resulted in four combinations of covariate outcome combinations (i) low care level - low professional experience, (ii) low care level - high professional experience, (iii) high care level - low professional experience, and (iv) high care level - high professional experience. Based on screening assessments, each household was classified into one of these four covariate combinations. The professional experience of 24h-caregivers was assessed with a newly developed scheme (supplementary table S1) that incorporates the duration of professional experience as well as the specific and general level of education. Two hundred (200) sets of three unique integer numbers per set (1, 2, 3) were generated by an online sequence generator tool. The numbers 1, 2, and 3 were replaced by the allocations (i) control, (ii) partial intervention, and (iii) full intervention, and consecutively listed under the four aforementioned covariate outcome combinations. Stratification criteria were assessed within the baseline survey. Households were then assigned in blocks of three. Group allocation was thus concealed at the time of the baseline assessment and revealed at the time of tablet dispensing preparation.

ASCOT score calculation details

The *ASCOT score* was calculated as 1-(x-1)*⅓, where "1-" turned the more positive outcome to a higher value, "x" represents the mean of seven items included in the index, "1" represents the smallest valid item value, and "⅓" represents the chosen maximum of the transformed index metric divided by the range of item values.

Efficacy score calculation details

The *Efficacy score* was calculated as (x-1)*¼, where the range of item values was 4, and cases were included if at least 10 out of 14 items were validly completed.

Table S1: Scoring scheme for professional experience (range: 0-5)

	**Professional experience (months)**		
**Education**	**0-5.99**	**6-11.99**	**12-**
No specific education (+1 for school leaving exam, i.e. ISCED 4, if professional experience is at least 6 m.)	0	1	2
Basic education: Caring Person, In-home Caregiver (+1 for school leaving exam, i.e. ISCED 4)	1	2	3
Basic and additional education(s) or Care Assistant (+1 for completed academic education, i.e. ISCED 6)	2	3	3
Graduate Nurse (+1 for completed academic education, i.e. ISCED 6)	3	4	4

Table S2. *ASCOT score* descriptive statistics (numeric details corresponding to figure 2)

	Baseline			Follow-up 1			Follow-up 2		
	mean	CI95	n	mean	CI95	n	mean	CI95	n
control	0.709	0.645, 0.772	34	0.692	0.615, 0.768	21	0.616	0.523, 0.708	14
partial intervention	0.745	0.701, 0.788	36	0.707	0.623, 0.792	21	0.745	0.619, 0.870	11
full intervention	0.687	0.631, 0.743	33	0.737	0.637, 0.836	15	0.752	0.628, 0.877	10

Table S3. *Efficacy score* descriptive statistics (numeric details corresponding to figure 3)

	Baseline			Follow-up 1			Follow-up 2		
	mean	CI95	n	mean	CI95	n	mean	CI95	n
control	0.916	0.876, 0.957	33	0.895	0.854, 0.935	22	0.839	0.758, 0.921	13
partial intervention	0.893	0.854, 0.931	33	0.919	0.870, 0.969	22	0.914	0.836, 0.992	11
full intervention	0.864	0.814, 0.914	32	0.908	0.791, 1.025	14	0.889	0.789, 0.989	10

Table S4. **Follow-up 1** (month 5) ANCOVA **ASCOT** (baseline value as covariate) comparison **control vs. any intervention**

	**control**			**any intervention**						
	mean	CI95	n	mean	CI95	n	F	η_p^2	p	adjusted p
**ASCOT score**	0.692	0.615, 0.768	21	0.720	0.659, 0.781	36	0.317	0.006	0.575	0.575
ASCOT “occupation”	1.92	1.61, 2.22	24	2.05	1.79, 2.31	37	0.470	0.008	0.496	0.496
ASCOT “control over daily life”	1.83	1.49, 2.16	23	2.05	1.80, 2.31	38	0.051	0.001	0.822	0.822
ASCOT “self-care”	1.96	1.64, 2.28	24	1.95	1.70, 2.19	37	0.079	0.001	0.779	0.779
ASCOT “personal safety”	1.42	1.17, 1.66	24	1.39	1.16, 1.63	38	0.036	0.001	0.851	0.851
ASCOT “social participation and involvement”	2.21	1.73, 2.69	24	1.71	1.44, 1.99	38	2.726	0.044	0.104	0.104
ASCOT “space and time to be yourself”	2.09	1.79, 2.39	22	2.03	1.80, 2.25	38	0.011	0.000	0.916	0.916
ASCOT “feeling encouraged and supported”	1.87	1.52, 2.22	23	1.89	1.89, 1.65	37	0.005	0.000	0.944	1.000

ASCOT score summarizes a mean score of 7 ASCOT for caregivers’ items, transformed to a scale ranging from 0 to 1, where 1 reflects the most positive outcome

Individual ASCOT items self-rated on a 4-point Likert-scale, where 1 is “ideal” and 4 indicates “high needs”

Overall ANCOVA: F(2)=2.248, p=0.116, partial eta²=0.078; n: control = 21, partial intervention = 21, full intervention = 15

Overall ANCOVA analyzed per intention to treat: F(2)=2.519, p=0.086, partial eta²=0.048, n: control = 34, partial intervention = 36, full intervention = 34

Covariate equal across groups assumption met: F(2)=1.200, p=0.306, partial eta²=0.023

Homogeneity of regression slopes assumption met (interaction term): F(2)=0.656, p=0.523, partial eta²=0.025

Normality assumption (score at m5) met: Control: sw(23)=0.930, p=0.107; Partial intervention: sw(23)=0.953, p=0.332; Full intervention: sw(17)=0.940, p=0.313

CI95 … 95% confidence interval, F … ANCOVA test statistic, η_p^2 … partial eta squared, p … ANCOVA derived p-value, ASCOT … Adult Social Care Outcomes Toolkit, sw … Shapiro-Wilk, adjusted p … Bonferroni-Holm adjusted p-value, adjusted for 2 contrasts (smaller p-value multiplied by 2)

Note: when running the ANCOVA with “nested data” added as covariate (12 nested households - nested means in this case that 2 participating 24h-caregivers working consecutively in the same household), test statistics for the *ACSOT score* are: F(2) = 0,223; p = 0,638.

Table S5. **Follow-up 1** (month 5) ANCOVA ASCOT (baseline value as covariate) comparison **partial vs. full intervention**

	**partial intervention**			**full intervention**						
	mean	CI95	n	mean	CI95	n	F	η_p^2	p	adjusted p
**ASCOT score**	0.707	0.624, 0.792	21	0.737	0.637, 0.836	15	4.868	0.129	**0.034**	0.068
ASCOT "occupation"	2.09	1.76, 2.42	22	2.00	1.53, 2.47	15	0.716	0.021	0.403	0.806
ASCOT "control over daily life"	2.09	1.74, 2.43	23	2.00	1.58, 2.42	15	0.002	0.000	0.962	1.000
ASCOT "self-care"	2.05	1.70, 2.39	22	1.80	1.43, 2.17	15	5.521	0.140	**0.025**	**0.050**
ASCOT "personal safety"	1.52	1.16, 1.89	23	1.20	0.97, 1.43	15	2.367	0.063	0.133	0.266
ASCOT "social participation and involvement"	1.65	1.32, 1.99	23	1.80	1.28, 2.32	15	0.209	0.006	0.651	1.000
ASCOT "space and time to be yourself"	2.13	1.83, 2.43	23	1.87	1.51, 2.22	15	4.368	0.111	**0.044**	0.088
ASCOT "feeling encouraged and supported"	1.91	1.58, 2.24	22	1.87	1.46, 2.28	15	0.700	0.020	0.409	0.409

ASCOT score summarizes a mean score of 7 ASCOT for caregivers' items, transformed to a scale ranging from 0 to 1, where 1 reflects the most positive outcome
Individual ASCOT items self-rated on a 4-point Likert-scale, where 1 is "ideal" and 4 indicates "high needs"
Overall ANCOVA: F(2)=2.248, p=0.116, partial eta²=0.078; n: control = 21, partial intervention = 21, full intervention = 15
Overall ANCOVA analyzed per intention to treat: F(2)=2.519, p=0.086, partial eta²=0.048, n: control = 34, partial intervention = 36, full intervention = 34
Covariate equal across groups assumption met: F(2)=1.200, p=0.306, partial eta²=0.023
Homogeneity of regression slopes assumption met (interaction term): F(2)=0.656, p=0.523, partial eta²=0.025
Normality assumption (score at m5) met: Control: sw(23)=0.930, p=0.107; Partial intervention: sw(23)=0.953, p=0.332; Full intervention: sw(17)=0.940, p=0.313
CI95 … 95% confidence interval, F … ANCOVA test statistic, η_p^2 … partial eta squared, p … ANCOVA derived p-value, ASCOT … Adult Social Care Outcomes Toolkit, sw … Shapiro-Wilk, adjusted p … Bonferroni-Holm adjusted p-value, adjusted for 2 contrasts (smaller p-value multiplied by 2)
Note: when running the ANCOVA with "nested data" added as covariate (12 nested households - nested means in this case that 2 participating 24h-caregivers working consecutively in the same household), test statistics for the *ACSOT score* are: F(2) = 4,692; p = 0,040.

Table S6. **Follow-up 2** (month 9) ANCOVA **ASCOT** (baseline value as covariate) comparison **control vs. any intervention**

	**control**			**any intervention**						
	mean	CI95	n	mean	CI95	n	F	η_p^2	p	adjusted p
**ASCOT score**	0.616	0.523, 0.708	14	0.748	0.668, 0.829	21	5.526	0.147	**0.025**	0.050
ASCOT "occupation"	2.33	1.93, 2.73	15	2.00	1.69, 2.31	22	2.143	0.059	0.152	0.304
ASCOT "control over daily life"	2.13	1.67, 2.60	15	2.04	1.64, 2.44	23	2.965	0.078	0.094	0.188
ASCOT "self-care"	2.27	1.73, 2.80	15	1.82	1.47, 2.17	22	3.594	0.096	0.066	0.132
ASCOT "personal safety"	1.60	1.32, 1.88	15	1.39	1.14, 1.64	23	0.761	0.021	0.389	0.778
ASCOT "social participation and involvement"	2.27	1.82, 2.71	15	1.83	1.49, 2.16	23	1.023	0.028	0.319	0.638
ASCOT "space and time to be yourself"	2.29	1.93, 2.64	14	2.00	1.68, 2.32	23	1.568	0.044	0.219	0.438
ASCOT "feeling encouraged and supported"	2.27	1.88, 2.66	15	1.68	1.40, 1.97	22	10.898	0.243	**0.002**	**0.004**

ASCOT score summarizes a mean score of 7 ASCOT for caregivers' items, transformed to a scale ranging from 0 to 1, where 1 reflects the most positive outcome
Individual ASCOT items self-rated on a 4-point Likert-scale, where 1 is "ideal" and 4 indicates "high needs"
Overall ANCOVA: F(2)=2.762, p=0.079, partial eta²=0.151, n: control = 14, partial intervention = 11, full intervention = 10
Overall ANCOVA analyzed per intention to treat: F(2)=3.143, p=0.047, partial eta²=0.059, n: control = 34, partial intervention = 36, full intervention = 34
Covariate equal across groups assumption met: F(2)=1.200, p=0.306, partial eta²=0.023
Homogeneity of regression slopes assumption met (interaction term): F(2)=1.279, p=0.294, partial eta²=0.081
Normality assumption (score at m9) met: Control: sw(14)=0.947, p=0.517; partial intervention: sw(13)=0.952, p=0.636; full intervention: sw(12)=0.943, p=0.534
CI95 … 95% confidence interval, F … ANCOVA test statistic, ηp² … partial eta squared, p … ANCOVA derived p-value, ASCOT … Adult Social Care Outcomes Toolkit, sw … Shapiro-Wilk, adjusted p … Bonferroni-Holm adjusted p-value, adjusted for 2 contrasts (smaller p-value multiplied by 2)
Note: when running the ANCOVA with "nested data" added as covariate (5 nested households - nested means in this case that 2 participating 24h-caregivers working consecutively in the same household), test statistics for the *ACSOT score* are: F(2) = 4,505; p = 0,042.

Table S7. **Follow-up 2** (month 9) ANCOVA **ASCOT** (baseline value as covariate) comparison **partial vs. full intervention**

	**partial intervention**			**full intervention**						
	mean	CI95	n	mean	CI95	n	F	η_p^2	p	adjusted p
**ASCOT score**	0.745	0.619, 0.870	11	0.752	0.628, 0.877	10	0.120	0.007	0.733	0.733
ASCOT "occupation"	2.00	1.53, 2.47	12	2.00	1.52, 2.48	10	0.002	0.000	0.968	0.968
ASCOT "control over daily life"	2.31	1.68, 2.93	13	1.70	1.22, 2.18	10	0.634	0.031	0.435	0.435
ASCOT "self-care"	1.92	1.34, 2.49	12	1.70	1.22, 2.18	10	0.357	0.018	0.557	0.557
ASCOT "personal safety"	1.38	0.99, 1.78	13	1.40	1.03, 1.77	10	0.052	0.003	0.823	0.823
ASCOT "social participation and involvement"	1.85	1.36, 2.33	13	1.80	1.24, 2.36	10	0.013	0.001	0.911	0.911
ASCOT "space and time to be yourself"	2.08	1.62, 2.54	13	1.90	1.37, 2.43	10	0.407	0.020	0.531	0.531
ASCOT "feeling encouraged and supported"	1.67	1.25, 2.08	12	1.70	1.22, 2.18	10	0.244	0.013	0.627	0.627

ASCOT score summarizes a mean score of 7 ASCOT for caregivers' items, transformed to a scale ranging from 0 to 1, where 1 reflects the most positive outcome
Individual ASCOT items self-rated on a 4-point Likert-scale, where 1 is "ideal" and 4 indicates "high needs"
Overall ANCOVA: F(2)=2.762, p=0.079, partial eta²=0.151, n: control = 14, partial intervention = 11, full intervention = 10
Overall ANCOVA analyzed per intention to treat: F(2)=3.143, p=0.047, partial eta²=0.059, n: control = 34, partial intervention = 36, full intervention = 34
Covariate equal across groups assumption met: F(2)=1.200, p=0.306, partial eta²=0.023
Homogeneity of regression slopes assumption met (interaction term): F(2)=1.279, p=0.294, partial eta²=0.081
Normality assumption (score at m9) met: Control: sw(14)=0.947, p=0.517; partial intervention: sw(13)=0.952, p=0.636; full intervention: sw(12)=0.943, p=0.534
CI95 … 95% confidence interval, F … ANCOVA test statistic, η_p^2 … partial eta squared, p … ANCOVA derived p-value, ASCOT … Adult Social Care Outcomes Toolkit, sw … Shapiro-Wilk, adjusted p … Bonferroni-Holm adjusted p-value, adjusted for 2 contrasts (smaller p-value multiplied by 2)
Note: when running the ANCOVA with "nested data" added as covariate (5 nested households - nested means in this case that 2 participating 24h-caregivers working consecutively in the same household), test statistics for the *ASCOT score* are: F = 0,024; p = 0,878.

Table S8. **Follow-up 1** (month 5) ANCOVA **efficacy survey** (baseline value as covariate) comparison **control vs. any intervention**

	**control**			**any intervention**						
	mean	CI95	n	mean	CI95	n	F	η_p^2	p	adjusted p
**Efficacy score**	0.895	0.85, 0.94	22	0.915	0.864, 0.966	36	0.771	0.014	0.384	0.768
Satisfaction with current docu	4.50	3.99, 5.01	20	4.68	4.37, 4.98	34	0.317	0.006	0.576	0.576
Completing docu is done quickly	4.65	4.27, 5.03	20	4.63	4.32, 4.95	30	0.014	0.000	0.906	0.906
Docu supports doing my job	4.15	3.62, 4.68	20	4.24	3.77, 4.71	33	0.054	0.001	0.818	1.000
I'm feeling competent in doing my job	4.78	4.51, 5.05	18	4.83	4.62.5.04	35	0.352	0.007	0.556	1.000
I'm well informed about care	4.58	4.12, 5.04	19	4.50	4.11, 4.89	34	0.077	0.002	0.782	1.000
I know the legal framework regarding my job	4.67	4.43, 4.91	18	4.79	4.56, 5.02	33	1.341	0.027	0.253	0.506
My German skills are adequate for doing my job	4.62	4.25, 4.99	21	4.51	4.21, 4.81	37	0.004	0.000	0.953	0.953
My professional skills are adequate for doing my job	4.58	4.12, 5.04	19	4.75	4.52, 4.98	36	3.648	0.066	0.062	0.124
I'm well prepared for emergencies	4.67	4.43, 4.91	18	4.81	4.59, 5.03	31	0.990	0.021	0.325	0.650
Docu provides safety	4.45	3.96, 4.94	20	4.66	4.39, 4.92	32	1.040	0.021	0.313	0.626
Docu provides knowledge on patient's condition	4.43	4.09, 4.77	21	4.74	4.52, 4.97	35	2.601	0.047	0.113	0.226
Networking	4.77	4.58, 4.96	22	4.55	4.13, 4.97	31	0.205	0.004	0.653	0.653
Availability contact persons	4.71	4.39, 5.04	21	4.68	4.35, 5.00	37	0.258	0.005	0.613	1.000
Communication with contacts	4.90	4.76, 5.04	20	4.86	4.67, 5.06	37	0.000	0.000	0.997	0.997

Efficacy score of 14 efficacy for 24h-caregivers' items, scored on a 5-point Likert scale, transformed to a scale ranging from 0 to 1, where 1 reflects the most positive outcome. Cases with at least 10 valid answers included (mean of valid answers calculated)

Overall ANCOVA: F(2)=0.417, p=0.661, partial eta²=0.015; n: control = 22, partial intervention = 22, full intervention = 14
Overall ANCOVA analyzed per intention to treat: F(2)=0.497, p=0.610, partial eta²=0.010, n: control = 30, partial intervention = 35, full intervention = 31
Covariate equal across groups assumption met: F(2)=1.546, p=0.218, partial eta²=0.032
Homogeneity of regression slopes assumption met (interaction term): F(2)=0.345, p=0.710, partial eta²=0.013
Normality assumption (score at m5) violated due to ceiling effects: Control: sw(23)=0.912, p=0.046; Partial intervention: sw(23)=0.744, p<0.001; Full intervention: sw(15)= 0.517, p<0.001
CI95 … 95% confidence interval, F … ANCOVA test statistic, η_p^2 … partial eta squared, p … ANCOVA derived p-value, ASCOT … Adult Social Care Outcomes Toolkit, sw … Shapiro-Wilk, adjusted p … Bonferroni-Holm adjusted p-value, adjusted for 2 contrasts (smaller p-value multiplied by 2)
Note: when running the ANCOVA with "nested data" added as covariate (12 nested households - nested means in this case that 2 participating 24h-caregivers working consecutively in the same household), test statistics for the *Efficacy score* are: F(2) = 0,510; p = 0,478.

Table S9. **Follow-up 1** (month 5) ANCOVA **efficacy survey** (baseline value as covariate) comparison **partial vs. full intervention**

	**partial intervention**			**full intervention**						
	mean	CI95	n	mean	CI95	n	F	η_p^2	p	adjusted p
**Efficacy score**	0.919	0.87, 0.97	22	0.908	0.791, 1.025	14	0.052	0.002	0.821	0.821
Satisfaction with current docu	4.81	4.50, 5.12	21	4.46	3.78, 5.14	13	1.533	0.047	**0.047**	0.094
Completing docu is done quickly	4.68	4.29, 5.08	19	4.55	3.92, 5.17	11	0.181	0.007	0.674	1.000
Docu supports doing my job	4.29	3.69, 4.88	21	4.17	3.27, 5.06	12	0.030	0.001	0.863	0.863
I'm feeling competent in doing my job	4.86	4.64, 5.07	21	4.79	4.32, 5.25	14	0.116	0.004	0.736	0.736
I'm well informed about care	4.50	4.03, 4.97	20	4.50	3.76, 5.24	14	0.000	0.000	0.997	0.997
I know the legal framework regarding my job	4.79	4.53, 5.05	19	4.79	4.32, 5.25	14	0.117	0.004	0.734	0.734
My German skills are adequate for doing my job	4.52	4.16, 4.89	23	4.50	3.91, 5.09	14	0.655	0.019	0.424	0.848
My professional skills are adequate for doing my job	4.74	4.47, 5.01	23	4.77	4.27, 5.27	13	0.164	0.005	0.688	0.688
I'm well prepared for emergencies	4.83	4.46, 5.02	18	4.77	4.27, 5.27	13	0.762	0.027	0.390	0.390
Docu provides safety	4.58	4.25, 4.91	19	4.77	4.27, 5.27	13	0.390	0.013	0.537	0.537
Docu provides knowledge on patient's condition	4.77	4.54, 5.01	22	4.69	4.18, 5.21	13	0.108	0.003	0.744	0.744
Networking	4.63	4.14, 5.12	19	4.42	3.54, 5.29	12	0.547	0.019	0.466	0.932
Availability contact persons	4.74	4.37, 5.11	23	4.57	3.90, 5.24	14	0.089	0.003	0.767	0.767
Communication with contacts	4.91	4.73, 5.09	23	4.79	4.32, 5.25	14	0.007	0.000	0.935	1

Efficacy score of 14 efficacy for 24h-caregivers' items, scored on a 5-point Likert scale, transformed to a scale ranging from 0 to 1, where 1 reflects the most positive outcome. Cases with at least 10 valid answers included (mean of valid answers calculated)

Overall ANCOVA: F(2)=0.417, p=0.661, partial eta²=0.015; n: control = 22, partial intervention = 22, full intervention = 14
Overall ANCOVA analyzed per intention to treat: F(2)=0.497, p=0.610, partial eta²=0.010, n: control = 30, partial intervention = 35, full intervention = 31
Covariate equal across groups assumption met: F(2)=1.546, p=0.218, partial eta²=0.032
Homogeneity of regression slopes assumption met (interaction term): F(2)=0.345, p=0.710, partial eta²=0.013
Normality assumption (score at m5) violated due to ceiling effects: Control: sw(23)=0.912, p=0.046; Partial intervention: sw(23)=0.744, p<0.001; Full intervention: sw(15)= 0.517, p<0.001
CI95 … 95% confidence interval, F … ANCOVA test statistic, η_p^2 … partial eta squared, p … ANCOVA derived p-value, ASCOT … Adult Social Care Outcomes Toolkit, sw … Shapiro-Wilk, adjusted p … Bonferroni-Holm adjusted p-value, adjusted for 2 contrasts (smaller p-value multiplied by 2)
Note: when running the ANCOVA with "nested data" added as covariate (12 nested households - nested means in this case that 2 participating 24h-caregivers working consecutively in the same household), test statistics for the *Efficacy score* are: F(2) = 0,062; p = 0,805.

Table S10. **Follow-up 2** (month 9) ANCOVA **efficacy survey** (baseline value as covariate) comparison **control vs. any intervention**

	**control**			**any intervention**						
	mean	CI95	n	mean	CI95	n	F	η_p^2	p	adjusted p
**Efficacy score**	0.839	0.76, 0.92	13	0.902	0.845, 0.959	21	7.868	0.202	**0.009**	**0.018**
Satisfaction with current docu	4.00	3.26, 4.74	11	4.68	4.43, 4.93	22	8.271	0.216	**0.007**	**0.014**
Completing docu is done quickly	4.08	3.25, 4.92	12	4.50	4.16, 4.84	16	0.839	0.032	0.369	0.738
Docu supports doing my job	3.64	2.67, 4.60	11	4.33	3.85, 4.82	18	3.880	0.130	0.060	0.120
I'm feeling competent in doing my job	4.83	4.47, 5.20	12	4.77	4.54, 5.01	22	0.170	0.005	0.683	0.683
I'm well informed about care	4.08	3.25, 4.92	12	4.50	4.18, 4.82	20	1.720	0.056	0.200	0.200
I know the legal framework regarding my job	4.90	4.67, 5.13	10	4.65	4.34, 4.96	20	0.063	0.002	0.803	0.803
My German skills are adequate for doing my job	4.57	4.20, 4.94	14	4.59	4.30, 4.89	22	0.268	0.008	0.608	0.608
My professional skills are adequate for doing my job	4.69	4.31, 5.07	13	4.81	4.58, 5.04	21	3.194	0.093	0.084	0.168
I'm well prepared for emergencies	4.64	4.30, 4.98	11	4.86	4.69, 5.02	21	4.000	0.121	0.055	0.110
Docu provides safety	4.08	3.28, 4.87	13	4.21	3.60, 4.82	19	0.343	0.012	0.563	1.000
Docu provides knowledge on patient's condition	4.29	3.67, 4.90	14	4.64	4.21, 5.06	22	1.990	0.057	0.168	0.336
Networking	4.77	4.50, 5.03	13	4.76	4.52, 5.01	21	0.572	0.018	0.455	0.455
Availability contact persons	4.57	4.08, 5.06	14	4.74	4.51, 4.97	23	0.769	0.022	0.387	0.774
Communication with contacts	4.43	3.69, 5.17	14	4.82	4.60, 5.04	22	2.371	0.067	0.133	0.266

Efficacy score of 14 efficacy for 24h-caregivers' items, scored on a 5-point Likert scale, transformed to a scale ranging from 0 to 1, where 1 reflects the most positive outcome. Cases with at least 10 valid answers included (mean of valid answers calculated)

Overall ANCOVA: F(2)=3.963, p=0.030, partial eta²=0.209; n: control = 13, partial intervention = 11, full intervention = 10
Overall ANCOVA analyzed per intention to treat: F(2)=1.839, p=0.165, partial eta²=0.038, n: control = 30, partial intervention = 36, full intervention = 32
Covariate equal across groups assumption met: F(2)=1.546, p=0.218, partial eta²=0.032
Homogeneity of regression slopes assumption met (interaction term): F(2)=0.002, p=0.998, partial eta²=0.000
Normality assumption (score at m9) violated due to ceiling effects: Control: sw(14)=0.950, p=0.557; Partial intervention: sw(13)=0.719, p<0.001; Full intervention: sw(11)= 0.787, p=0.006
CI95 … 95% confidence interval, F … ANCOVA test statistic, η_p^2 … partial eta squared, p … ANCOVA derived p-value, ASCOT … Adult Social Care Outcomes Toolkit, sw … Shapiro-Wilk, adjusted p … Bonferroni-Holm adjusted p-value, adjusted for 2 contrasts (smaller p-value multiplied by 2)
Note: when running the ANCOVA with "nested data" added as covariate (5 nested households - nested means in this case that 2 participating 24h-caregivers working consecutively in the same household), test statistics for the *Efficacy score* are: F(2) = 5,311; p = 0,028.

Table S11. **Follow-up 2** (month 9) ANCOVA **efficacy survey** (baseline value as covariate) comparison **partial vs. full intervention**

	**partial intervention**			**full intervention**						
	mean	CI95	n	mean	CI95	n	F	η_p^2	p	adjusted p
**Efficacy score**	0.914	0.836, 0.992	11	0.889	0.789, 0.989	10	0.285	0.016	0.600	0.600
Satisfaction with current docu	4.75	4.36, 5.14	12	4.60	4.23, 4.97	10	0.080	0.004	0.781	0.781
Completing docu is done quickly	4.63	4.00, 5.25	8	4.38	3.94, 4.81	8	0.315	0.026	0.564	0.564
Docu supports doing my job	4.40	3.71, 5.09	10	4.25	3.38, 5.12	8	0.066	0.004	0.800	0.800
I'm feeling competent in doing my job	4.85	4.61, 5.07	13	4.67	4.12, 5.21	9	0.468	0.024	0.502	1.000
I'm well informed about care	4.75	4.46, 5.04	12	4.13	4.43, 4.82	8	1.861	0.099	0.190	0.380
I know the legal framework regarding my job	4.91	4.71, 5.11	11	4.33	3.67, 5.00	9	3.613	0.074	0.074	0.148
My German skills are adequate for doing my job	4.67	4.25, 5.08	12	4.50	3.99, 5.01	10	0.287	0.015	0.598	1.000
My professional skills are adequate for doing my job	4.75	4.36, 5.14	12	4.89	4.63, 5.15	9	0.000	0.000	1.000	1.000
I'm well prepared for emergencies	4.83	4.59, 5.08	12	4.89	4.63, 5.15	9	0.005	0.000	0.947	0.947
Docu provides safety	4.33	3.56, 5.10	9	4.10	3.01, 5.19	10	0.013	0.001	0.911	0.911
Docu provides knowledge on patient's condition	4.83	4.47, 5.20	12	4.40	3.50, 5.30	10	0.056	0.003	0.815	0.815
Networking	4.64	4.18, 5.09	11	4.90	4.67, 5.13	10	2.256	0.111	0.150	0.300

Availability contact persons	4.77	4.50, 5.03	13	4.70	4.22, 5.18	10	0.478	0.023	0.497	0.497
Communication with contacts	4.83	4.59, 5.08	12	4.80	4.35, 5.25	10	0.572	0.029	0.459	0.459

Efficacy score of 14 efficacy for 24h-caregivers' items, scored on a 5-point Likert scale, transformed to a scale ranging from 0 to 1, where 1 reflects the most positive outcome. Cases with at least 10 valid answers included (mean of valid answers calculated)
Overall ANCOVA: F(2)=3.963, p=0.030, partial eta²=0.209; n: control = 13, partial intervention = 11, full intervention = 10
Overall ANCOVA analyzed per intention to treat: F(2)=1.839, p=0.165, partial eta²=0.038, n: control = 30, partial intervention = 36, full intervention = 32
Covariate equal across groups assumption met: F(2)=1.546, p=0.218, partial eta²=0.032
Homogeneity of regression slopes assumption met (interaction term): F(2)=0.002, p=0.998, partial eta²=0.000
Normality assumption (score at m9) violated due to ceiling effects: Control: sw(14)=0.950, p=0.557; Partial intervention: sw(13)=0.719, p<0.001; Full intervention: sw(11)= 0.787, p=0.006
CI95 … 95% confidence interval, F … ANCOVA test statistic, η_p^2 … partial eta squared, p … ANCOVA derived p-value, ASCOT … Adult Social Care Outcomes Toolkit, sw … Shapiro-Wilk, adjusted p … Bonferroni-Holm adjusted p-value, adjusted for 2 contrasts (smaller p-value multiplied by 2)
Note: when running the ANCOVA with "nested data" added as covariate (5 nested households - nested means in this case that 2 participating 24h-caregivers worked consecutively in the same household), test statistics for the *Efficacy score* are: F(2) = 0,457; p = 0,508.

Table S12. Pearson's correlations of baseline data with follow-ups for variance inspection for *ASCOT score* and *Efficacy score*

	Follow-up 1			Follow-up 2		
	r	p	n	r	p	n
ASCOT score	0.540	<0.001	57	0.701	<0.001	35
Efficacy score	0.543	<0.001	58	0.642	<0.001	34

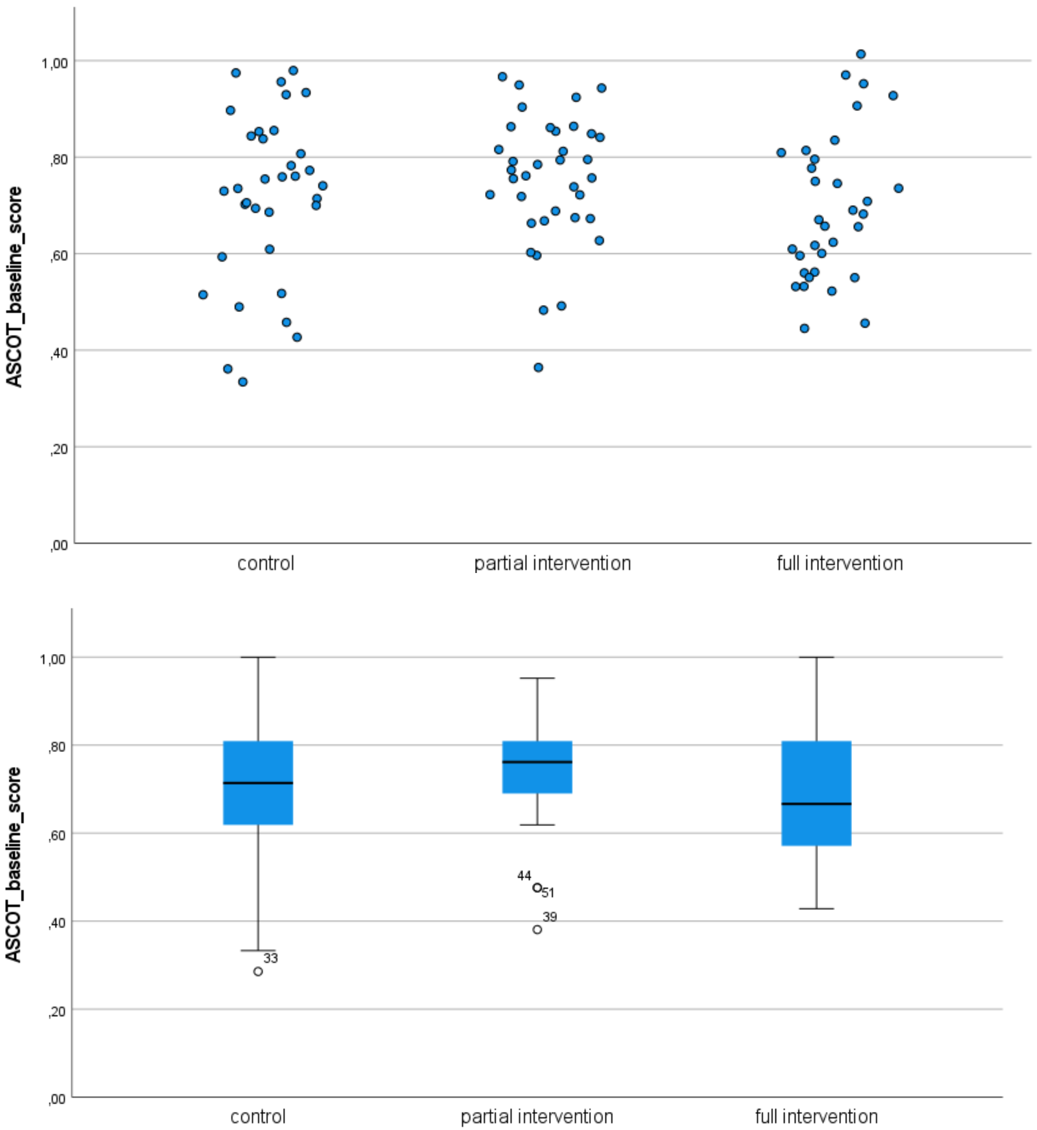

Figure S1: Beeswarm plot and corresponding boxplots for the ***ASCOT score*** at **baseline**

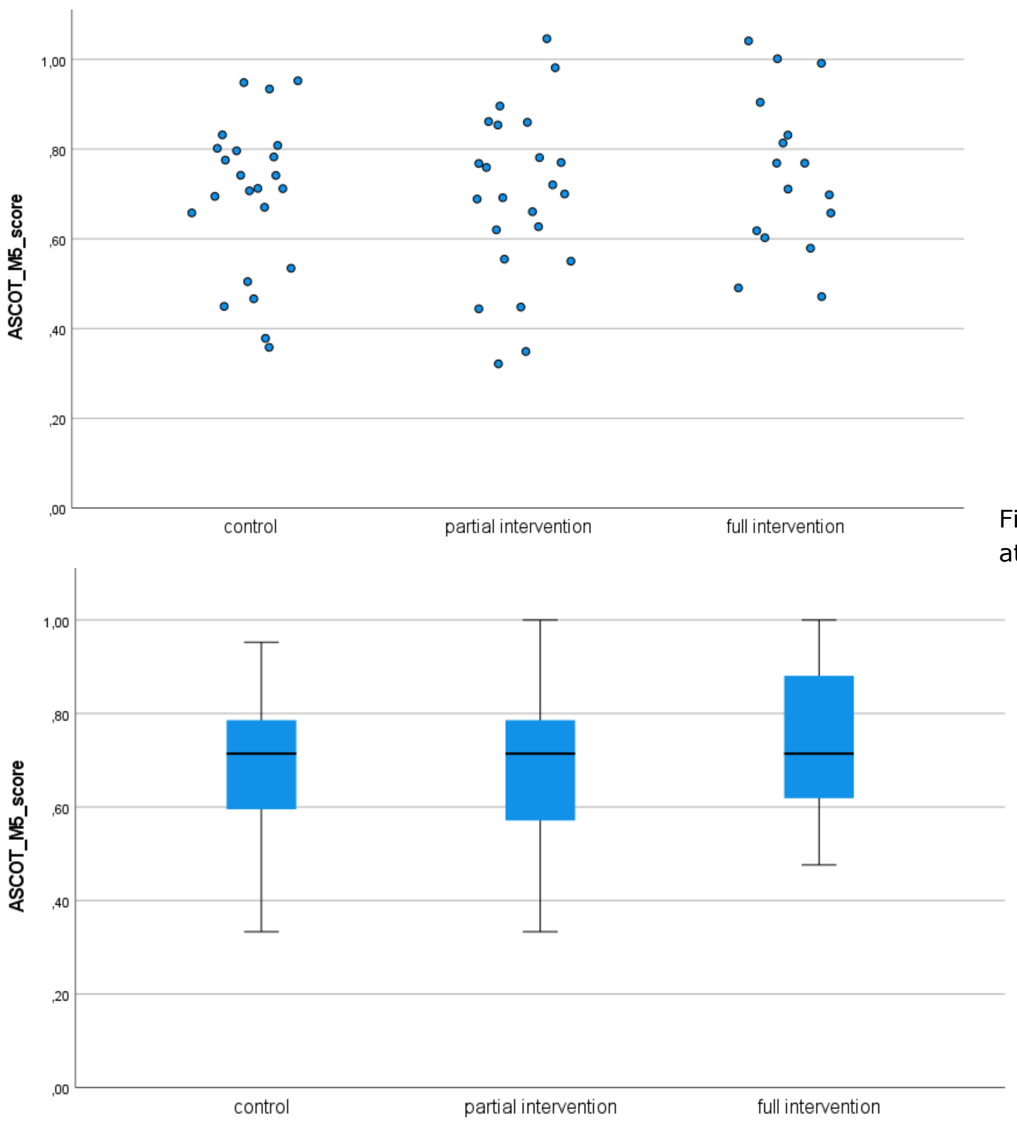

Figure S2: Beeswarm plot and corresponding boxplots for the ***ASCOT score*** at the **5-month follow-up**

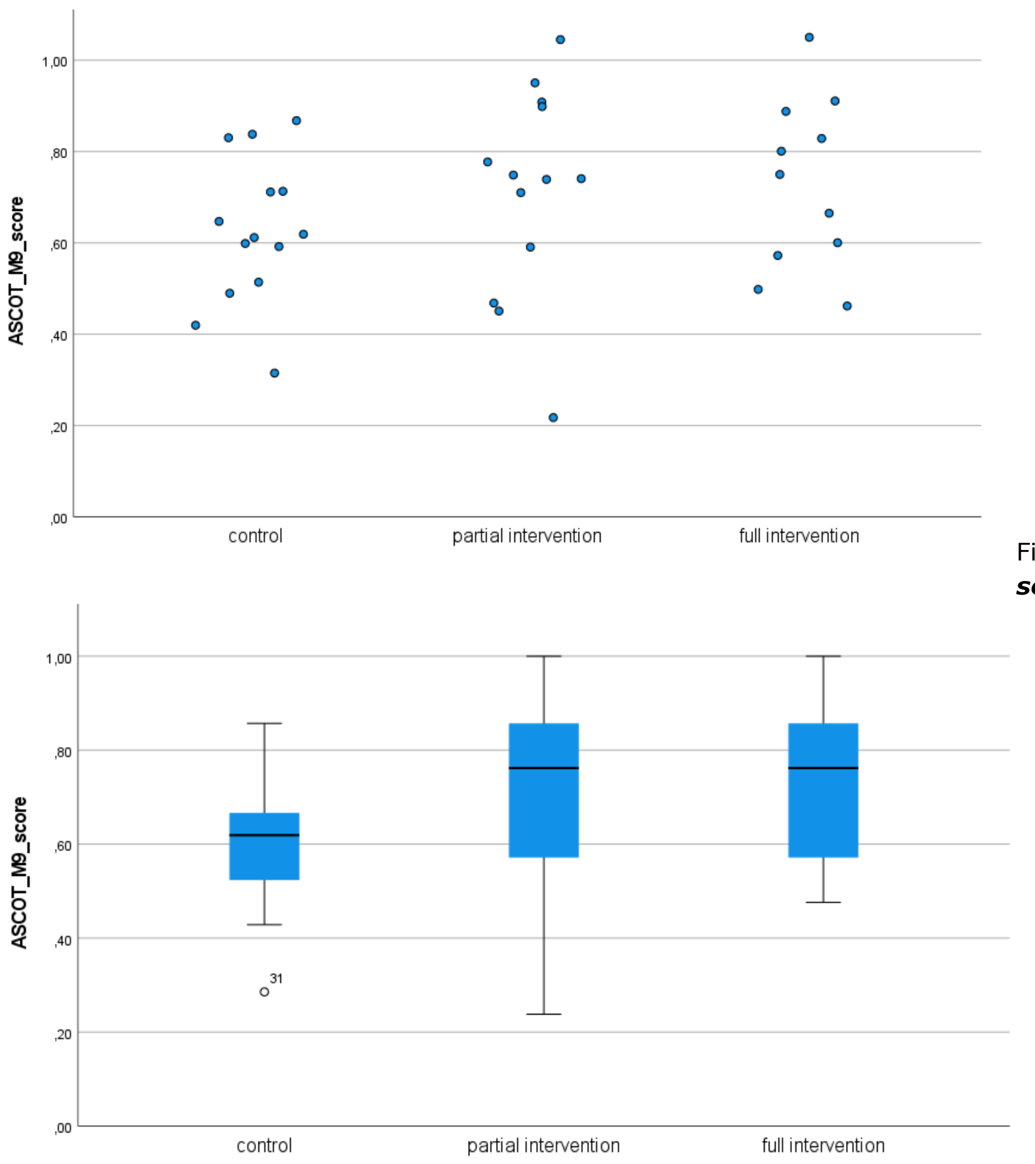

Figure S3: Beeswarm plot and corresponding boxplots for the ***ASCOT score*** at the **9-month follow-up**

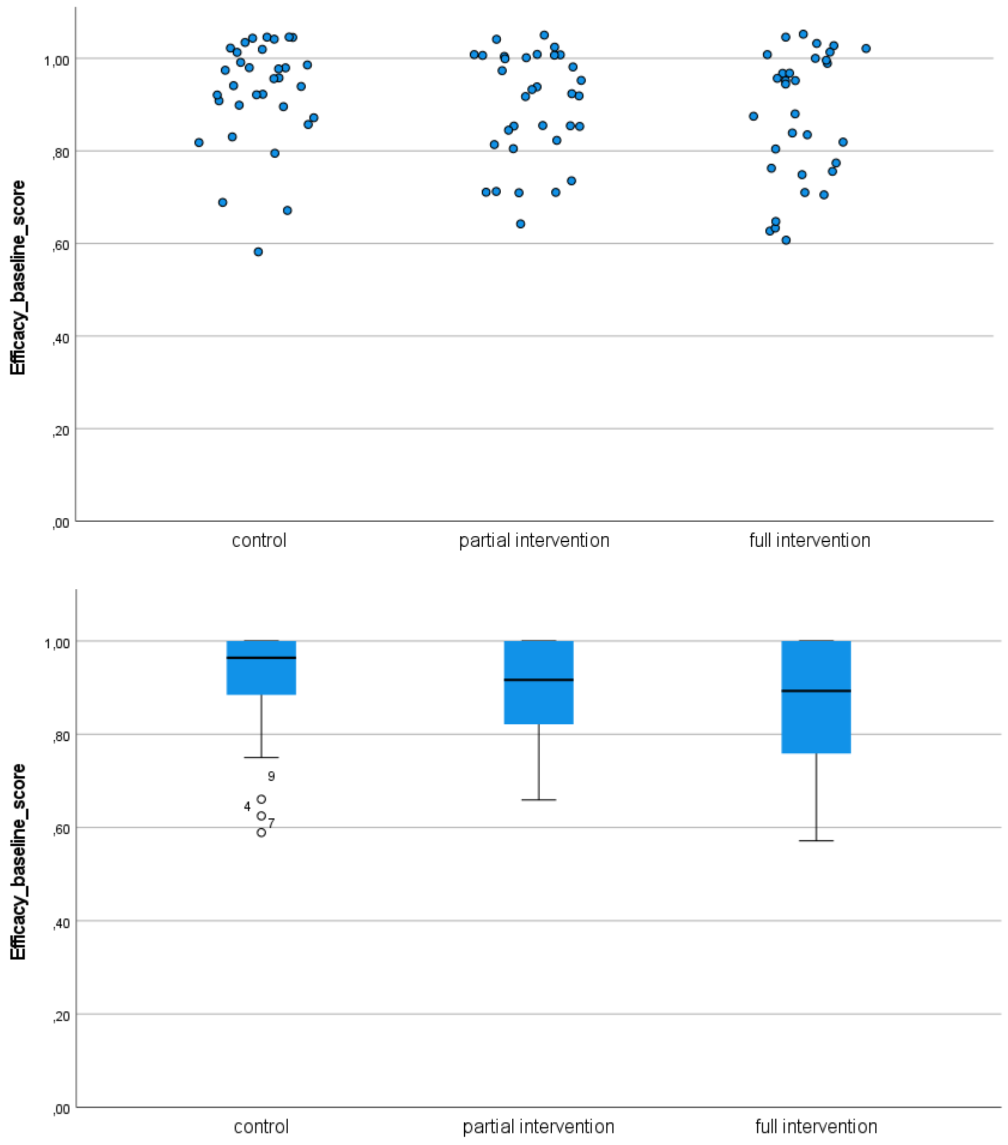

Figure S4: Beeswarm plot and corresponding boxplots for the ***Efficacy score*** at **baseline**

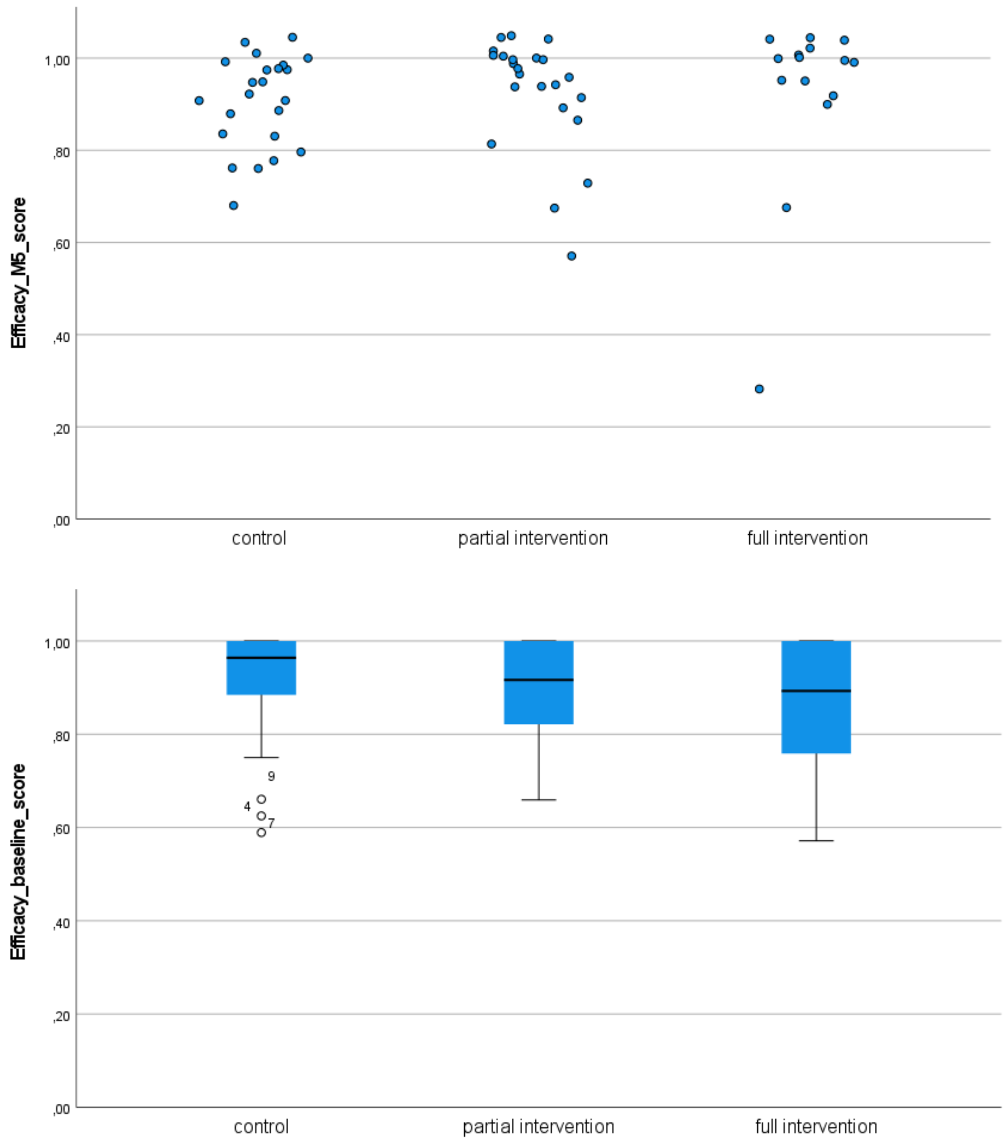

Figure S5: Beeswarm plot and corresponding boxplots for the ***Efficacy score*** at the **5-month follow-up**

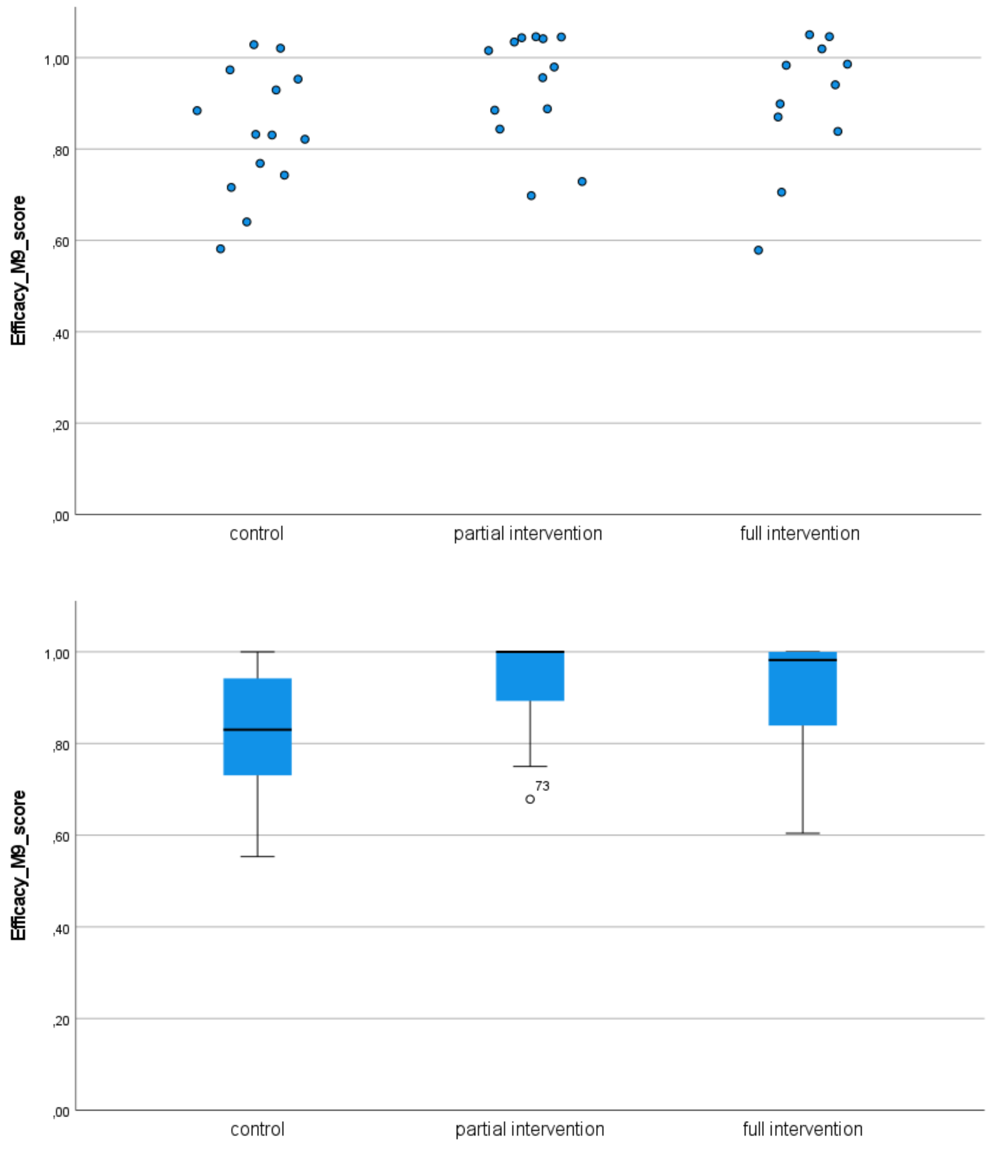

Figure S6: Beeswarm plot and corresponding boxplots for the ***Efficacy score*** at the **9-month follow-up**
